# Smartphone Technology to Empower People Experiencing Homelessness: Secondary Analysis

**DOI:** 10.2196/27787

**Published:** 2021-09-29

**Authors:** Whitney Thurman, Monika Semwal, Leticia R Moczygemba, Mark Hilbelink

**Affiliations:** 1 School of Nursing University of Texas at Austin Austin, TX United States; 2 Health Outcomes Division College of Pharmacy University of Texas at Austin Austin, TX United States; 3 Sunrise Homeless Navigation Center Austin, TX United States

**Keywords:** homelessness, self-management, smartphone technology, social needs, mobile phone

## Abstract

**Background:**

In the United States, the number of people experiencing homelessness has continually increased over the last 3 years. Homelessness is associated with poor health, and people experiencing homelessness are often burdened with high rates of chronic and mental health conditions, functional limitations, and cognitive impairment. Despite the high burden of chronic illness and functional limitations, there is limited literature exploring self-management among homeless populations.

**Objective:**

This study aims to investigate how access to smartphone technology facilitates self-management, including the attainment of social needs within the context of homelessness.

**Methods:**

A secondary analysis of 33 exit interviews from 2 feasibility studies related to mobile health interventions among people experiencing homelessness was conducted. Iterative thematic analysis was used to identify themes representative of participants’ experiences using smartphone technology.

**Results:**

Collectively, participants revealed not only how the context of homelessness constrained their ability to engage in activities necessary to self-manage health and meet social needs but also how consistent and predictable access to the tools available through a smartphone changed their behaviors and outlook. The global theme of *empowered by technology* was identified and defined as how having a smartphone with a plan for unlimited text, calling, data, and transportation allowed participants to navigate homelessness and facilitated self-management.

**Conclusions:**

People experiencing homelessness used the tools on a smartphone to make decisions, take action, solve problems, and use the resources—skills necessary for fulfilling tasks required for effective self-management. Further, consistent access to smartphone technology and transportation empowered participants to meet the requirements for the attainment of social needs.

## Introduction

### Background

In the United States, the number of people experiencing homelessness has continually increased over the last 3 years, with a 3% increase from 552,830 in 2018 to 567,715 in 2019 [1]. This number is likely to increase during the ongoing COVID-19 pandemic [2]. Homelessness is associated with poor health, and people experiencing homelessness are often burdened with high rates of chronic and mental health conditions, functional limitations, and cognitive impairment [3-5]. Furthermore, the rates of disability are high among people experiencing homelessness. In 2016, almost 50% of people experiencing homelessness had some type of disability compared with 20% of the general US population [6].

The reasons for poor health are multifactorial and include risk factors that increase the risk of homelessness in the first place, such as early life poverty, mental illness, and substance use disorders. However, many risk factors are secondary to the conditions of homelessness, such as poor nutrition, exposure to communicable diseases, harsh living conditions, and violence [7,8]. Many people experiencing homelessness face difficulties in connecting to and accessing appropriate health and social services because of social and psychological barriers such as stigma, lack of trust in health care providers, and transient lifestyles [9-11]. Furthermore, people experiencing homelessness struggle to meet the basic needs such as food, shelter, and transportation, resulting in self-management of chronic health conditions being overlooked or neglected [12-14]. These barriers and competing priorities contribute to adverse outcomes, including reliance on emergency departments (EDs) or hospitals for nonemergent health care services and increased risk of 30-day hospital readmissions [15-17].

Self-management has been defined as “the ability of the individual, in conjunction with family, community, and health care professionals, to manage symptoms; treatments; lifestyle changes; and psychosocial, cultural, and spiritual consequences of health conditions” [18] and requires patients to perform a variety of day-to-day tasks to manage symptoms, treatments, and consequences of chronic conditions [19]. A component of self-management also includes developing the skills needed to devise, implement, evaluate, and revise plans to take charge of one’s own health [20]. Given that self-management requires planning ahead, dedicated resources, and access to information, it is not surprising that homelessness poses unique barriers to successful self-management. For example, people experiencing homelessness have described poor sleeping conditions, limited food choices, limited income, and a lack of access to storage as barriers to self-management [21].

Despite the high burden of chronic illness and functional limitations, there is limited literature exploring self-management among homeless populations. To the extent that self-management has been investigated among people experiencing homelessness, outcomes are encouraging [22-24]. In addition, Merdsoy et al [21] explored the perceptions, needs, and preferences for self-management among men experiencing homelessness. They found that the majority of participants perceived self-management as important, but complex challenges related to homelessness, including lack of social support and limited access to communication technology, dictated unique self-management support needs [21].

Given the distinct context of homelessness and the importance of social support and access to information and communication for effective self-management, smartphone technology may be a useful tool to facilitate self-management activities among people experiencing homelessness. In the United States, smartphones are ubiquitous. For people experiencing homelessness who often encounter many barriers to communication and information seeking, smartphones allow for multiple resources in one tool, helping overcome access barriers. Prior evidence suggests that the connectivity provided by smartphones can decrease social isolation and facilitate communication between peers, family, and service providers [25]. In addition, pilot studies have investigated using phone calls for improving medication adherence [26], apps for improving mental health [27], and text messaging for appointment reminders [28] and improving physical activity [29] among people experiencing homelessness. This burgeoning body of evidence suggests that people experiencing homelessness are welcoming and supportive of using smartphone technology to address their health care issues, and smartphone technology has also been identified as a feasible tool for self-management support among people experiencing homelessness [25].

### Objective

The purpose of this study is to investigate how access to smartphone technology facilitates self-management, including the attainment of social needs within the context of homelessness. The methods and results are reported in accordance with the Consolidated Criteria for Reporting Qualitative studies checklist [30].

## Methods

### Overview

This study is a secondary analysis of exit interviews from 2 pilot studies investigating the preliminary outcomes of two mobile health interventions among people experiencing homelessness [25]. Both studies were conducted in Austin, Texas, United States. The first pilot study was a 4-month study designed to improve care coordination and reduce acute health care utilization among people experiencing homelessness [25]. The second was a 1-month study designed to improve medication adherence among people experiencing homelessness. In each study, participants were recruited from 3 churches that provided services to people experiencing homelessness. Convenience sampling was used, and potential participants were referred by community partner site staff or volunteers to the research team for study eligibility and screening (for eligibility criteria for each study, refer to Textbox 1). At baseline, the first author (WT) administered a series of baseline assessments to collect demographic data and self-reported health history, medication adherence, social support, and recent hospitalizations or ED visits. Upon enrollment into the study, participants received a study-assigned smartphone activated with a plan for unlimited text, calling, data, and public transportation.

Exit interviews were conducted between February and June 2019 with 16 people experiencing homelessness who participated in the first study and between August and September 2019 with 17 people experiencing homelessness who participated in the second study. The semistructured interviews included questions about participating in the study, having and using a smartphone, ED or hospital visits during the study period, and medication use. The interviews allowed participants to share their experiences with smartphone technology and how access to a smartphone with a data plan and access to unlimited public transportation affected their daily lives and routines. For this secondary analysis, investigators focused on the component of the interview in which participants described how they used their smartphones to accomplish various daily tasks. Interviews were conducted by the first author (WT) in quiet, private locations in church-based entities from which the participants had been recruited. Interviews were digitally recorded, and a team of graduate students transcribed and verified each interview. Both studies were approved by the university institutional review board, and informed consent was obtained before participant enrollment.

**Inclusion criteria for research studies.**

**Study 1**

- Aged ≥18 years
- Currently experiencing homelessness (defined as where the person had slept most nights in the previous 30 days)
- Currently prescribed at least two medications for chronic health conditions (self-report)
- Diagnosis of at least one chronic health condition (self-report)
- At least two hospitalizations or emergency department visits in the past 6 months (self-report)
- Score of at least 10 on the Patient Health Questionnaire-9

**Study 2**

- Aged ≥18 years
- Currently experiencing homelessness (defined as where the person had slept most nights in the previous 30 days)
- Currently prescribed at least two medications for chronic health conditions (self-report)
- Diagnosis of at least one chronic health condition (self-report)
- At least two hospitalizations or emergency department visits in the past 6 months (self-report)
- Score of at least 4 on the Rapid Estimate of Adult Literacy in Medicine-Short Form

### Data Analysis

#### Overview

Iterative thematic analysis situated within a critical realism framework [31] was used to explore how participants incorporated smartphone technology into their lives and how the broader social context of homelessness influenced their experiences with technology and their interpretations of those experiences [32]. Three authors (WT, MS, and LRM) of this paper independently read 3 transcripts to familiarize themselves with the data before independently conducting line-by-line coding and then meeting to discuss the initial themes. After 2 subsequent rounds of independent coding and discussion, the team reached consensus on the themes and finalized the codebook. Once consensus regarding the codebook was reached, the remaining transcripts were divided between the 3-member coding team to independently code, and iterative analyses continued until saturation of major themes was achieved. Dedoose software (SocioCultural Research Consultants) was used to facilitate coding and data management.

#### Qualitative Rigor

Transferability was addressed by providing detailed descriptions of data and context of homelessness and reporting data using participants’ direct quotes. Credibility of data analysis was enhanced by having each of the first 3 authors (WT, MS, and LRM) critically analyze the data before reflecting on the analytic process. This process enabled the authors to discuss negative cases, which facilitated a thick description of the data [33]. Reflexivity was maintained through ongoing documentation in a research log, comparing these notes against the data and engaging in whole-group discussions about the reflections.

### Sample

A total of 31 unique individuals are represented in the 33 interviews. On average, participants were 42.7 (SD 9.67) years and had been experiencing homelessness for a mean of 7.4 years (range 1 month to 30 years). Of the 31 participants, 25 (81%) were unsheltered, sleeping on the street or in tents, whereas 4 (13%) reported staying in a shelter, 1 (3%) had just been released from jail, and 1 (3%) reported staying with a friend. Participants reported a substantial burden of chronic illness as 77% (24/31) experienced multiple chronic conditions (≥2) and 55% (17/31) reported being prescribed at least four different medications. Participant demographics and health-related characteristics are given in Table 1.

**Table 1:** Participant demographics (N=31).

Demographic variable		Values
Age (years), mean (SD)		42.7 (1.73)
Length of homelessness (years), mean (SD)		8.1 (7.7)
**Gender, n (%)**		
	Male	21 (68)
	Female	7 (23)
	Transgender female	1 (3)
	Transgender male	1 (3)
	Other	1 (3)
**Race and ethnicity, n (%)**		
	White and non-Hispanic	21 (68)
	Black and non-Hispanic	5 (16)
	Hispanic	2 (6)
	Asian	1 (3)
	Other	2 (6)
**Functional limitations, n (%)**		
	0	11 (36)
	1	9 (29)
	2	7 (23)
	3+	4 (13)
**Chronic conditions, n (%)**		
	Hypertension	20 (64)
	High cholesterol	10 (32)
	Asthma	9 (29)
	COPD^a^	9 (29)
	Diabetes mellitus	8 (26)
	Anxiety or depression	7 (23)
	Bipolar disorder	5 (16)
	Seizure disorder	5 (16)
	Brain injury	4 (13)
	Myocardial infarction or stroke	2 (6)
	Multiple chronic conditions (≥2)	24 (77)
**Prescribed medications, n (%)**		
	2-3	14 (45)
	4-5	7 (23)
	6+	10 (32)

^a^COPD: chronic obstructive pulmonary disease.

Just over half of the participants (16/31, 52%) reported having a personal cell phone with service upon enrollment in the study. Of those with phones at baseline, the majority (14/16, 87%) reported that their phones were capable of text messaging, picture messaging, and mobile apps use. Of the 48% (15/31) of the participants who did not have a personal cell phone at baseline, 93% (14/15) reported that they did not have a cell phone because it was expensive, 13% (2/15) reported having no use of a phone, 7% (1/15) reported not knowing how to use a phone, and 27% (4/15) reported concerns about privacy.

## Results

### Overview

The qualitative analysis identified two major themes and eight subthemes that captured the ways in which smartphone technology facilitated self-management within the social context of homelessness. Collectively, participants revealed not only how the *context of homelessness* constrained their ability to engage in activities necessary to self-manage health and meet social needs but also how consistent and predictable access to the tools available through the smartphone changed their behaviors, thoughts, and outlook toward daily life. Thus, the global theme of *empowered by technology* was identified and defined as how having the smartphone activated with a plan for unlimited text, calling, data, and public transportation allowed participants to navigate the context of homelessness and facilitated self-management. Textbox 2 includes representative quotes from each of the themes and subthemes.

**Themes and representative quotes.**

**Context of homelessness**

- Being stigmatized
  “The first time I had a stroke, it wasn’t diagnosed. I was put in a cab and sent to my room.”
  “...a lot of businesses don’t allow people—homeless people—to come in and charge their phones. They cut the outlets off.”
- Encountering barriers
  “I mean it’s hard to try and get bus passes, when-especially when you’re broke. I mean, you either have to make deals with people who trade them for cigarettes or dollar here, dollar there. Or you got to get up and go to churches like super early in the morning where they hand them out.”
  “I been sitting three weeks for this guy just to bring me two letters. Two letters so I could return it to the people from [apartment complex] trying to get me an apartment. I am still sitting there, so I guess I get up early tomorrow and go knock on his office door.”
- Precarious environments
  “Why would I want someone to find me? Well, anything could happen. Get sick. I could get you know beat up, passed out somewhere. No one knows where I am, then the phone helps find me.”
  “Cause being on the streets and homeless, you know, every day is a matter of survival.”
- Symptom experience
  “Like over the last two days, my breathing has been so bad I’ve nearly gone to the emergency room. Over the last 3 days actually. Every day I’d be thinking about it. I need to go, I need to go. My breathing’s really bad. I didn’t, but it’s just hit or miss for me whether I do or not.”
  “I was hit by a van and run over. I already had a hernia pretty bad...and when I got hit by the van, it tore the skin open and parts of my small intestines was coming out. I ignored it for about two months and the infection got so bad, the pain was so intense that I was blacking out in pain so I had to head to the ER.”
- Resilience
  “...no we’re not that dumb...we’re really smart because we have to go through all this stuff to still be living.”

**Empowered by technology**

- Connecting with others
  “I had a phone number people could reach me at, so that was definitely a good, good thing.”
  “the new phone is so amazing. It will take you around the world.”
- Regaining control
  “It’s like a life assistant.”
  “This phone actually helps me out though cause I can contact people that I need to talk to on a daily basis and get the information done and get situations taken care of.”
- Getting things done
  “phones are so important to have nowadays. You really have to have one to be able to keep track of everything.”

### Context of Homelessness

#### Overview

The majority of participants had multiple morbidities and were actively seeking to manage their health. However, the context of homelessness often precluded participants from successfully doing so. Participants described factors directly related to lack of housing and safe shelter that included being stigmatized, encountering barriers such as antiloitering policies and complex bureaucracies to access health care and social services, living in precarious environments, and experiencing symptoms such as disabling pain that contributed to their inability to successfully manage their health. Participants also revealed resilience that allowed them to reconcile their current circumstances with hope for a better future.

#### Being Stigmatized

Participants uniformly reported being treated differently—usually poorly—by members of the community during daily interactions and by health care professionals during clinical encounters. One participant described an ongoing battle with a gastric ulcer stemming from his physician refusing to prescribe antibiotics out of the fear that the participant’s homelessness would prevent him from finishing the entire regimen. Another participant shared his experience of asking a front desk clerk at a downtown hotel to call an ambulance for him during an emergency health situation:

They wouldn’t call 911. They would not call me an ambulance...instead, they called security on me because I was homeless.

#### Encountering Barriers

In addition to the differential treatment they received, the stigma experienced by participants also created barriers that prevented them from accessing needed goods and services and forced their attention away from higher-order tasks needed for self-management. For example, participants had to carefully plan their days to complete relatively mundane tasks such as charging their cellphones. Participants described difficulty in keeping their phones charged because of many businesses instituting antiloitering policies that require individuals to purchase a product to use electrical outlets. Thus, participants learned which businesses would allow them to access electrical outlets and strategized to ensure that they could safely spend time, often spending hours, each day doing so.

Another frequently encountered barrier was the inability to maintain contact with important others, including family, friends, and service providers. Participants shared how the lack of a mailing address created barriers to, for example, receiving important communications regarding disability benefits or receiving a government-issued cell phone. They also cited the lack of a phone number where they could be reached as a substantial impediment to obtaining employment and housing. One participant succinctly stated this barrier when he said as follows:

You know, my only other option would have been to give them the phone number for the [shelter], which is a message phone to a homeless shelter, you know. Not fun convincing an employer to hire a homeless guy.

#### Precarious Environments

Instability is a hallmark characteristic of homelessness, and participants clearly articulated the precarious environments in which they found themselves. Participants lived in a state of constant worry about their personal safety and the safety of their possessions. One participant said:

people that may be so desperate to make a few bucks off of something they see that phone and they maybe follow you or do things to try obtain that phone including, but not limited to-to you know threatening your own health.

Given the precarity of their situations, participants struggled to assert or maintain control of their daily lives, possessions, and health. Participants often had to rely on others to meet their needs and described the goodwill of bus drivers who sometimes let them ride for free and the willingness of some service providers to bend rules on their behalf. Participants also described being taken advantage of, especially because they lacked options.

#### Symptom Experience

The context of homelessness also defined participants’ symptom experience, influencing not only the perception and evaluation of symptoms but also the management of such experiences. Participants often attributed their current homelessness to symptoms stemming from their chronic health conditions. One participant clearly explained this as follows:

I am homeless because I had...four brain surgeries. I mean, I was fine. I had a home and everything...Now I lost everything.

Another participant described his reliance on medication after experiencing a brain injury when he was younger:

how important it is for me...to be on medicine that allows me to function or do stuff cause otherwise I can’t keep a job, I can’t keep a house, I can’t you know.

Pain was a nearly universal symptom. One participant described how he had experienced so much pain for so long that he considered his pain to be a part of him. Participants also described the creative ways in which they managed their symptoms while experiencing homelessness. One participant who suffered from chronic pain stemming from injuries incurred during his time spent fighting in Afghanistan described how carrying a large backpack full of his personal belongings doubled as therapeutic as it helped to compress his back and alleviate some of his lower back pain.

Anxiety and depression were also widespread, and participants described various ways in which they managed their symptoms. When asked how she prevented herself from succumbing to misery, one participant simply said *drugs*. She went on to recount how she would prefer a prescription medication that allows her to function, but in lieu of that, she uses small amounts of illicit substances to help her overcome her anxiety, focus on tasks she needs to do, and engage with other people. Importantly, participants recognized that prescribed medications can interact with alcohol and other substances and took steps to manage potential adverse outcomes.

#### Resilience

Participants also revealed a certain resilience that allowed them to persevere despite the stigmatizing attitudes, barriers, precarious environments, and unrelenting symptoms of chronic illness and homelessness. This means participants were able to cope with the context of homelessness by relying on their personal ingenuity and determination and on their social connections, however fragile they may have been. One participant described installing solar panels at his camp, providing him with electricity and the benefits associated with it.

Although other participants had more straightforward strategies for navigating their circumstances, participants’ collective fortitude was revealed succinctly by one participant who had moved into housing during the study time frame when she said, “when I was living on the streets, I always found a way.”

### Empowered by Technology

#### Overview

Despite the circumstances of homelessness, participants persevered in efforts to meet their health and social needs and experienced varying degrees of success. However, when provided with a smartphone activated with a plan for unlimited text, calling, and data as well as access to public transportation, participants demonstrated how these basic tools equipped them to more easily navigate the context of homelessness. During exit interviews, participants revealed how they went above and beyond the expectations of the research studies and incorporated the technology into their daily lives. This consistent access to the tools provided by the phone as well as the information and resources available through the internet caused a shift in participants’ behaviors, thoughts, and perceptions, resulting in participants being empowered by the technology and, subsequently, better able to engage in self-management activities. Specifically, the subthemes that support this global theme are *connecting with others, getting things done,* and *regaining control*.

#### Connecting With Others

A dedicated phone number and consistent phone service facilitated participants’ ability to stay connected with family and friends and, in some instances, served as a catalyst for reconnection after long periods of no contact. One participant shared how he was able to talk with his daughter and hear the cry of his first grandchild over the phone. A different participant tearfully recounted saying goodbye to his mother who fell ill and died suddenly during the course of the research study. He said:

I would have never gotten to say goodbye to my mom if I didn’t have that phone.

For some participants, the mobile bus pass and resultant access to transportation was as important for connecting with others as was the ability to make phone calls. Participants described traveling to other parts of the city to talk with friends and acquaintances that they had not seen or communicated with in a while. Conversely, some participants availed themselves of their transportation options to leave social situations that they judged to be unsafe or unhealthy. The phone and access to transportation also facilitated participants’ ability to reciprocate social support. For example, one participant took the opportunity to teach his peers who were also participants how to navigate the bus system and locate various food pantries in town.

Importantly, the tools available via smartphones also allowed participants to maintain connections with service providers, including case managers, physicians, and others involved in helping them navigate the complex world of homeless services. One participant who moved into housing during the course of the study counted 4 case managers with whom he worked. He said as follows:

I had so many appointments and doctor visits and this and that and the other. So the phone was integral. I would not be in that place [housing] I can guarantee right now if I did not have that phone.

Transportation access also allowed participants to more easily travel to appointments, facilitating both their ability to adhere to health advice and some relief to their health symptoms. Indeed, being able to navigate the urban landscape via bus instead of foot resulted in less pain and easier breathing for many participants who struggled to manage chronic respiratory conditions.

#### Getting Things Done

Getting things done was defined as using the phone for tasks or activities that went above and beyond the requirements or expectations of the research studies. Given access to the tools available through smartphones, participants were able to accomplish relatively mundane tasks that were nearly impossible to do without these tools. For example, participants described being able to call providers and schedule appointments. One participant succinctly stated:

I am able to talk to people that I need to talk to like social security, the food stamp place, and I can get doctors’ appointments squared away with it and when I wasn’t doing that before...it helps me to be more alert and it helps me to take care of my business...when I need to because everything is on the phone.

Having a phone meant that not only did they have access to information and to others, but they also always knew what time it was, which allowed them to establish routines that facilitated self-management. Participants described using prompts and time schedules to get "day-to-day stuff done" and how "a bus pass ready to go...any type of information that is needed on the information highway...[and] entertainment" available at their fingertips kept them moving toward their goals.

This ability to get things done by using the tools available on the smartphone facilitated the attainment of several social needs among participants enrolled in the 4-month study. Specifically, 6 participants moved into housing, 5 gained employment, and 1 obtained disability benefits during their time in the study, and participants credited their access to both the study phone and reliable transportation with their ability to maintain contact and attend appointments that were required to attain these social needs.

#### Regaining Control

Ultimately, access to the tools available on the smartphone, including transportation and internet access, allowed participants to regain control over aspects of their environments and their everyday lives. For example, one participant said as follows:

It’s too hot so I stayed on. I mean, I just always had a place to go with that bus pass. I didn’t have to beg the bus driver to let me on.

Another participant described the phone thusly, “...it gave me a little bit more extra freedom and kept me reminded of things I needed to do and stuff and things I wanted to do.”

Others described shifting perceptions of themselves and their circumstances that empowered them to reassert control. One participant described how, by virtue of the smartphone and consistent access to transportation, he was able to think beyond his own survival needs and how this shifted his perceptions of his circumstances. He described the experience of having a smartphone and participating in the research study as helping him get back on track and said:

makes you feel a little bit more important, like I mean especially out on the street it makes you feel like you’re doing something. Makes you feel a little bit better than what you are out here.

He credited his decision to re-establish a connection with his case manager and enroll in a temporary housing program to this shift in his self-perception.

Participants also attributed the tools made available via the smartphone with decreasing their worry and improving their general sense of well-being:

It was a godsend. It really was. I mean because I didn’t have to worry about a lot of things. I could make phone calls when I needed to...It just took a lot of burden off me knowing that I had a bus pass. I had a phone I could use, you know, if I got in trouble or something or was in a bad situation and so it was very helpful for that.

## Discussion

### Principal Findings

Findings from this study extend our understanding of how mobile technology—specifically, the predictable and consistent access to the tools available on a smartphone—can facilitate self-management, including attainment of social needs within homeless populations. Smartphones allowed participants to have multiple resources, including internet access, mobile apps, email, maps, and entertainment, on one device that overcomes barriers to storing and keeping track of multiple items. This robust functionality, as compared with older phones without these features, is important to facilitate the connection between people experiencing homelessness and their communities.

Effective self-management requires individuals to manage the symptoms and consequences of chronic illness by regular monitoring and management of physical, cognitive, behavioral, and emotional changes [19]. Lorig and Holman [34] suggested that these activities can be classified as medical, emotional, and role management tasks, and our findings indicate that access to unlimited data, text, calling, and transportation can support people experiencing homelessness to successfully engage in these activities. This means that participants revealed how they were able to use the tools on the smartphone to make decisions, take action, solve problems, and use resources—skills necessary for fulfilling the tasks required for self-management and meeting social needs [34].

A hallmark characteristic of self-management is a collaborative approach in which patients partner with professionals to devise strategies related to the ongoing management of symptoms and illness [35] This type of patient-provider partnership enables decision-making and problem solving, but these activities are predicated on having access to appropriate information. Thus, without consistent and reliable access to communication technologies, it is nearly impossible for people experiencing homelessness to engage in this collaborative approach because of an inability to maintain contact with providers or access the required information and resources. However, our findings support earlier evidence suggesting that people experiencing homelessness perceive self-management as important as participants in this study sought to be active participants in their health care and proactively sought information about their health conditions and engaged with providers when possible [21]. Thus, when equipped with smartphone technology, participants were empowered to actively engage in self-management tasks and activities.

Unfortunately, without a phone, many people experiencing homelessness simply miss opportunities for housing and employment because they cannot be located in a timely manner and do not have reliable contact information for an employer. At least six participants stayed engaged with case managers via the smartphone to complete the requirements to get into housing during the course of the study, and five participants reported gaining employment. This indicates that a smartphone is a powerful and necessary tool for people experiencing homelessness to meet social needs.

It should be noted that the context of homelessness presents challenges for everyday use of technology. Similar to prior research, participants in this study indicated that maintaining charged devices and keeping devices protected from the elements and safe from others require ongoing efforts [36]. Participants described several methods used to navigate these circumstances to charge their phones and access the tools available to them. However, developing and implementing those strategies was time-consuming and inefficient. Furthermore, in this study, some participants mentioned that being responsible for the study-assigned smartphone caused added worries in their daily lives. These barriers suggest that community partners and social service providers should consider allowing people experiencing homelessness to spend time in their facilities safely charging their devices.

In addition to connecting them with professionals, access to the tools on the smartphone also facilitated connections to friends and family. Emotional management is an essential component of self-management [34], and this connection to others enables social support and reciprocal relationships that not only support self-management but also enhance resilience [37]. Earlier evidence has identified the mental strength that people experiencing homelessness with chronic illness need to survive while living on the street [38], and our findings indicate that connection to others can enhance resilience and mental strength in this population. Specifically, findings from this study extend our understanding of resilience in the presence of adversity and how equipping people with proper tools can return a sense of agency and autonomy—both of which are needed for successful self-management. Indeed, the accomplishment of the daily tasks described in this study transcended the study expectations, indicating that people experiencing homelessness are motivated and ready to *do what needs to be done* to self-manage their lives. Thus, an important implication of the current work is the need to use asset-based approaches when working with homeless populations. Evidence reveals that people experiencing homelessness value health and understand the importance of self-management [21,22], and the resilience of people experiencing homelessness has been acknowledged by prior authors [39,40]. Capitalizing on these assets by equipping homeless populations with consistent and reliable tools for communication, access to information, and transportation could yield important benefits, including a sense of security and autonomy that can empower people experiencing homelessness to navigate the context of homelessness and reassert control over their health and lives.

Findings from this study also suggest that access to the tools and information available via smartphones serve more practical purposes as well. Participants were more easily able to access food and other humanitarian aid satisfying daily subsistence needs and, subsequently, increasing their capacity to pursue higher-order tasks, including scheduling and attending appointments with health and social services providers. Gobeil-Lavoie et al [41] identified that one of the main challenges to self-management is the prioritization of self-care and that patients with multiple conditions must choose which care activities to prioritize. They also found that most individuals with multiple chronic conditions identify a single disorder on which to focus their efforts and that social and economic conditions will influence how patients prioritize self-care [41]. Our participants were no different and reported multiple competing demands on their time. Similarly, prior research has identified a prevailing present time orientation among people experiencing homelessness, the unpredictability of time while experiencing homelessness, and a sense of not having enough time [42], precluding the ability of many individuals in this population to meet the expectations of our health and social services systems as they are currently structured. Indeed, most participants described how consistently knowing the time of day helped them create routines in a way that was not possible without the phone. Many also used the tools on smartphones such as alarms for reminders to take medications and manage appointments.

### Limitations

There are limitations to this study that should be considered. First, participants in both of these studies were recruited from church-based locations. It is possible that people experiencing homelessness who access services from community-based locations such as churches may have different needs or different abilities than individuals who access services from more traditional locations such as clinics or shelters. In addition, the relative success that the participants had with managing their smartphones, including keeping them safe, keeping them charged, and using them to manage everyday tasks, may indicate that this sample is more cognitively intact than many people experiencing homelessness. Thus, the findings of this study should be interpreted with caution.

### Conclusions

Homelessness is a complex problem that presents barriers to the provision of appropriate health and social services, and people experiencing homelessness must navigate complex bureaucratic systems while also managing complex comorbidities without much, if any support to do so. Resolving homelessness and providing appropriate services will require multifaceted systems approaches achieved, largely, through policy change. However, findings from this analysis reveal that individual-level interventions also play an important role in building capacity among people experiencing homelessness. Indeed, our findings indicate that equipped with the basic tools of smartphone technology and reliable access to transportation, people experiencing homelessness can and do engage in activities needed to self-manage their health and meet social needs. As communities across the United States and globally define and incorporate system-wide strategies to prevent and end homelessness, the relatively straightforward and comparatively inexpensive tools of smartphone technology and public transportation are smart investments to help individuals navigate homelessness and self-manage their own health and social needs. Our findings indicate that access to communication, information via the internet, and transportation facilitates the use of these higher-order skills to self-manage their health and attain social needs.

## Abbreviations

ED: emergency department
